# TOTAL OXIDANT AND ANTIOXIDANT LEVELS IN PATIENTS WITH GALLBLADDER STONES OR RELATED COMPLICATIONS: ARE THEY IMPORTANT FOR TREATMENT?

**DOI:** 10.1590/0102-6720202400043e1837

**Published:** 2024-12-02

**Authors:** Muhammed Emin ÇELIK, Veysel Garani SOYLU, Ayse YILMAZ

**Affiliations:** 1 Ankara Bilkent City Hospital, Intensive Care Unit – Ankara, Türkiye; 2 Kastamonu University, Faculty of Medicine, Intensive Care Unit – Kastamonu, Türkiye; 3 Kastamonu University, Faculty of Medicine, Anesthesiology and Reanimation Unit – Kastamonu, Türkiye.

**Keywords:** Oxidants, Gallstones, Free radicals, Oxidantes, Cálculos biliares, Radicais livres

## Abstract

**BACKGROUND:**

Many free radicals result in an inflammatory process due to complications caused by gallstones. These free radicals are inactivated by various reactions and participate in different reactions. Molecules are oxidants and antioxidants that take an active role in almost every event that takes place in the body.

**AIMS:**

To analyse the changes in total antioxidant level (TAL) and total oxidant level (TOL) in the follow-up of patients hospitalized for cholelithiasis or its complications, showing the active oxidative stress, and to test the usability of these parameters in the evaluation of treatment success.

**METHODS:**

Forty-five patients took part in the study. Blood samples were taken twice, previous to surgery and 6 hours after surgery. Tissue samples were also obtained from patients who were operated. Then, the samples were sent to a laboratory to measure the total oxidant and antioxidant status of patients.

**RESULTS:**

The median for the TAL_before (pre-operation or hospitalization in non-operational) variable was 2.40 (interquartile range — IQR=0.50), and the median for the TAL_after variable was 2.20 (IQR=0.33). The median of the tissue-derived TAL variable was 0.32 (IQR=0.13), and the median of the TOL variable was 0.43 (IQR=0.52). The median value of the TAL_before variable for men was 2.50 (IQR=0.50), while the median value for the TAL_before variable for women was 2.30 (IQR=0.50). TAL_before variable values did not show a statistically significant difference according to gender (Z=1.446; p=0.154, p>0.05). Similarly, the median values of TOL_before variable by gender were similar (Z=0.614; p=0.545, p>0.05).

**CONCLUSIONS:**

Cholelithiasis and its complications cause many inflammatory responses, ending with free radical formation. During follow-up, its level decreases due to consumption or success of the treatment.

## RESUMO

**RACIONAL:**

Muitos radicais livres resultam do processo inflamatório devido a complicações causadas por cálculos biliares. Esses radicais livres são inativados por várias reações e participam de diferentes reações. As moléculas são oxidantes e antioxidantes, que desempenham um papel ativo em quase todos os eventos que ocorrem no corpo.

**OBJETIVOS:**

Analisar as alterações do nível de antioxidante total (NAT) e no nível oxidante total (NOT) no acompanhamento de pacientes hospitalizados por colelitíase ou suas complicações, evidenciando o estresse oxidativo ativo e testar a usabilidade desses parâmetros na avaliação do sucesso do tratamento.

**MÉTODOS:**

Foram incluídos no estudo 45 pacientes. Amostras de sangue foram coletadas duas vezes antes da cirurgia e 6 horas após a cirurgia. Além disso, foram coletadas amostras de tecido de pacientes operados. Em seguida, foram enviados ao laboratório para medir os níveis total de oxidantes e antioxidantes.

**RESULTADOS:**

A mediana da variável NAT antes (pré-operatório ou internação em não-operatório) foi 2,40 (IIQ=0,50), e a mediana da variável NAT depois foi 2,20 (IIQ=0,33). A mediana da variável NAT derivada de tecido foi 0,32 (IQR=0,13) e a mediana da variável NOT foi 0,43 (IQR=0,52). O valor mediano da variável NAT antes para os homens foi 2,50 (IIQ=0,50), enquanto o valor mediano da variável NAT antes para as mulheres foi 2,30 (IIQ=0,50). Os valores da variável NAT antes não apresentaram diferença estatisticamente significativa segundo o sexo (Z=1,446; p=0,154, p>0,05). De forma similar; os valores medianos da variável NOT antes, por sexo, foram semelhantes (Z=0,614; p=0,545, p>0,05).

**CONCLUSÕES:**

A colelitíase e suas complicações causam diversas respostas inflamatórias, culminando com a formação de radicais livres. Durante o tratamento, seu nível diminui conforme o consumo ou o sucesso do tratamento.

## INTRODUCTION

Gallstones and their complications are very common and occupy an important place in hospitalizations.There are many publications in the literature on the formation mechanisms of gallstones^18,19^. In complications caused by them, many free radicals are formed in the inflammatory process^17^. These free radicals are inactivated by various reactions and participate in different reactions. Molecules are oxidant and antioxidant and take an active role in almost every event that takes place in the body.

It has taken years for the importance of these molecules to be fully grasped and understood. In recent years, after it was understood that these molecules have an important place in managing body functions, cellular defense, and even aging, various attempts have been made to make quantitative measurements of these values^1,11^.

Many studies have been conducted on how these molecules change in conditions such as angina, small vessel occlusion, and respiratory failure^7^. In the studies, it was concluded that total antioxidant level (TAL) and total oxidant level (TOL) measurements are more effective in cases where the catabolic phase is more active. Subsequently, the use of regular changes in blood values of these levels gained prominence in terms of assessing the success of treatment^4^.

Hospitalizations due to gallbladder disease are very common in general surgery clinics. There may be patients who are operated on simultaneously with hospitalization, and some patient groups are followed with conservative treatment rather than acute surgery. Some events mentioned above increase oxidative stress in both patient groups, although for different reasons^3^.

The study aims to show the active oxidative stress and to test the usability of these parameters in the evaluation of treatment success. In this study, the changes in TAL and TOL were evaluated in the follow-up of patients hospitalized for cholelithiasis or its complications.

## METHODS

The study is a prospective observational study. Forty-five patients admitted to Atatürk Training and Research Hospital with cholelithiasis or its complications were included in the study. Written informed consent was obtained from the patients participating in the study. All procedures were carried out according to the Declaration of Helsinki and ethical rules, and the study was approved by the Ethics Committee of the Institution (number 745566).

### Operation procedure

Venous blood values, C-reactive protein (CRP) values, and blood gas parameters of the patients were determined and lactate values were recorded. Oxidative and antioxidative parameters such as TAL, TOL, arylesterase (ARES), total thiol levels (TTL), paraoxonase (PON), and stimulated paraoxonase (SPON) were analyzed from the blood samples of the patients. The blood samples were taken from the patients upon admission to the service. After they were centrifuged, they were kept at -80°C until they were examined. Tissue samples from the sac fundus were taken from those operated patients and sent to the laboratory to be studied. Tissues were kept at -80°C until studied. On the study day, the tissues were dissolved and weighed 200 mg each and homogenized in 2 mL of physiological saline. For homogenization, the tissues were first mechanically lysed at moderate speed for about 5 minutes. Then homogenization was carried out with an ultrasonic wave homogenizer for 60 seconds. After the mixtures were centrifuged at 10,000 g, the supernatant was separated and biochemical measurements were made with these samples.

All chemicals used are of analytical grade and were obtained from Merck and Sigma. Type 1 distilled water was used in the preparation of the solutions.

#### Devices used

- Reagents were weighed with a Mettler Toledo ML 204/01 precision scale (Switzerland).

- IKA RCT basic (Germany) device was used as a magnetic stirrer.

- The pH of the buffers was measured with a INOLAB pH730 (Germany) pH meter.

- Mechanical homogenization was done with a T18B, IKA (Germany) instrument.

- Ultrasonic homogenization was done with a UW mini 20 SONOPLUS, BANDELIN (Germany) instrument.

- Automated TTL, TAL, TOL, PON, SPON, and ARES tests were released in the Cobas 6000 c501, ROCHE (Japan) module.

### Statistical analysis

The Statistical Package for the Social Sciences (SPSS) for Windows 15.0 (SPSS Inc., Chicago, ILL, USA) program was used. The statistical significance level was accepted as p<0.05.

The conformity of the variables of age, TAL (before, after), TOL (before, after), ARES (before, after), CRP, operation time, etc. to the normal distribution in the study was evaluated using the Shapiro-Wilk test.

Mean±standard deviation descriptive statistics were used for the normally distributed age variable. Median (interquartile range — IQR) and minimum-maximum descriptive statistics were used for non-normally distributed variables such as operative time and ARES.

Number (n) and percentage values were used for categorical variables such as gender, operation, and American Society of Anesthesiologists (ASA) score obtained within the scope of the study.

The Mann-Whitney U test was used to determine whether TAL before and TOL before variable values differed according to gender and determined age group, and TAL after, TOL after, etc. variable values differed according to operation time groups. Similarly, the Mann-Whitney U test was used to compare variable values such as TAL before, TOL before, ARES before, CRP and lactate in those who had surgery and those who could not.

Wilcoxon Signed Rank Test was used to evaluate whether preoperative and postoperative TAL, TOL, ARES, and oxygenated total thiol levels (OXTTL) values and variable values such as TAL, TOL, and ARES obtained from blood and tissue differed.

## RESULTS

The mean age of the patients was 50.78±14.56 years. Of the 45 patients included in the study, 22.2% (n=10) were men and 77.8% (n=35) were women. There were 34 (75.6%) patients who underwent surgery and 11 (24.4%) patients who could not be operated (Table 1).

**Table 1:** Distribution of individuals by gender, surgery status, American Society of Anesthesiologists scores and type of operation.

	n (%)
Gender	
Male	10 (22.2)
Woman	35 (77.8)
Surgery status	
Operated	34 (75.6)
Non-operated	11 (24.4)
ASA	
1	25 (55.6)
2	19 (42.2)
3	1 (2.2)
Type of operation (n=34)	
Lap	32 (94.2)
Lap+Conversion	1 (2.9)
Conversion	1 (2.9)

ASA: American Society of Anesthesiologists; Lap: laparoscopy.

The median for the TAL before (pre-operation or hospitalization in non-operational) variable was 2.40 (IQR=0.50), and the median for the TALafter variable was 2.20 (IQR=0.33) (Table 2). The median operative time was 40.0 (IQR=15.0) min.

**Table 2:** Descriptive statistics of variables obtained from patients.

	Nº	Min-Max	Median (IQR)
TAL before	45	1.90–3.20	2.40 (0.50)
TAL after	34	1.70–2.90	2.20 (0.33)
TOL before	45	0.30–23.30	3.90 (3.80)
TOL after	34	0.20–6.50	2.30 (2.36)
PON before	45	47.30–357.40	144.90 (145.80)
PON after	34	44.90–287.90	128.75 (101.63)
SPON before	45	87.50–1,071.50	437.50 (495.30)
SPON after	34	79.70–902.90	372.95 (368.38)
ARES before	45	30.30–137.90	78.00 (42.25)
ARES after	34	13.90–124.10	69.85 (42.60)
OXTTL before	45	34.00–235.70	120.10 (63.10)
OXTTL after	34	54.10–238.90	119.35 (56.15)
Surgery time(minutes)	34	20.0–150.0	40.0 (15.0)
CRP	45	0.12–35.80	0.84 (3.04)
Lactate	44	6.0–29.0	16.0 (5.0)

Min: minimum; Max: maximum; IQR: Interquartile Range; TAL: total antioxidant level; TOL: total oxidant level; PON: Paraoxonase; SPON: stimulated paraoxonase; ARES: Arylesterase; OXTTL: oxygenated total thiol levels; CRP: C-reactive protein.

The median of the tissue-derived TAL variable was 0.32 (IQR=0.13), and the median of the TOL variable was 0.43 (IQR= 0.52) (Table 3). It was determined that the gamma-glutamyl transferase (GGT) median was 15.51 (IQR=14.89) and the TTL median was 118.45 (IQR=31.86).

**Table 3:** Descriptive statistics for the variables obtained from the tissue (n=34).

	Min-Max	Median (IQR)
TAL	0.13–1.30	0.32 (0.13)
TOL	0.06–1.46	0.43 (0.52)
PON	0.05–1.71	0.42 (0.40)
SPON	0.23–7.83	3.17 (2.92)
ARES	3.32–64.29	25.72 (13.10)
GGT	5.15–53.57	15.51 (14.89)
TTL	61.93–275.63	118.45 (31.86)

Min: minimum; Max: maximum; IQR: Interquartile Range; TAL: total antioxidant level; TOL: total oxidant level; PON: Paraoxonase; SPON: stimulated paraoxonase; ARES: Arylesterase; GGT: gamma-glutamyl transferase; TTL: Total thiol levels.

The median value of the TAL before variable for men was 2.50 (IQR=0.50), while the median value for the TAL before variable for women was 2.30 (IQR=0.50) (Table 4). TAL before variable values did not show a statistically significant difference according to gender (Z=1.446; p=0.154, p>0.05). Similarly, median values of TOL before variable by gender were similar (Z=0.614; p=0.545, p>0.05).

**Table 4:** Comparison of total antioxidant level and total oxidant level values by gender.

	Male (n=10)		Female (n=35)		Z	p
	Min-Max	Median (IQR)	Min-Max	Median (IQR)		
TAL before	2.10–2.90	2.50 (0.50)	1.90–3.20	2.30 (0.50)	1.446	0.154
TO Lafter	0.80–6.80	3.69 (4.30)	0.30–23.30	4.23 (3.80)	0.614	0.545

Min: minimum; Max: maximum; IQR: Interquartile Range; TAL: total antioxidant level; TOL: total oxidant level.

The median age of individuals before TAL is 2.30 (IQR=0.20) years for those aged 40 years and younger, and 2.40 (IQR=0.50) years for those over 40 years (Table 5). It was determined that the TAL before and TOL before values obtained in the age groups did not differ statistically significantly (Z=1.204; p=0.732, p>0.05 and Z=0.236; p=0.470, p>0.05, respectively).

**Table 5:** Comparison of total antioxidant level before and total oxidant level before values according to the determined age groups.

	Age				Z	p
	<40 year (n=12)		>40 year (n=33)			
	Min-Max	Median (IQR)	Min-Max	Median (IQR)		
TAL before	1.90–3.10	2.30 (0.20)	2.00–3.20	2.40 (0.50)	1.204	0.732
TOL before	0.30–23.30	4.18 (9.30)	0.70–23.30	3.90 (3.60)	0.236	0.470

Min: minimum; Max: maximum; IQR: Interquartile Range; TAL: total antioxidant level; TOL: total oxidant level.

The TAL before and TOL before values of the participants in the study did not differ significantly according to the operation status (Z=0.067, p=0.948, p<0.05 and Z=0.423; p=0.687, p>0.05 respectively). The median TAL before surgery was 2.40 (IQR=0.43) and the median TAL before surgery was 2.30 (IQR=0.70) for those who did not have surgery (Table 6).

**Table 6:** Comparison of the variable values specified according to the state of being operated.

	Surgery status				Z	p
	Surgery (n=34)		Nonsurgery (n=11)			
	Min-Max	Median (IQR)	Min-Max	Median (IQR)		
TAL before	1.90–2.90	2.40 (0.43)	2.00–3.20	2.30 (0.70)	0.067	0.948
TOL before	0.30–23.30	4.17 (4.09)	0.80–12.10	3.56 (2.99)	0.423	0.687
PON before	47.30–357.40	140.60 (112.78)	65.30–299.60	224.00 (161.60)	0.396	0.706
SPON before	87.50–1071.50	414.80 (376.03)	155.00–951.80	674.40 (533.30)	0.581	0.575
ARES before	35.50–137.90	78.90 (38.78)	30.30–136.20	69.20 (64.40)	0.819	0.426
OXTTL before	53.20–235.70	130.50 (57.33)	34.00–149.30	94.00 (67.10)	2.760	0.005
CRP	0.12–8.79	0.48 (1.26)	0.67–35.80	7.78 (19.84)	3.697	<0.001
LACTATE	9.0–29.0	16.0 (5.0)	6.0–25.0	15.0 (9.0)	0.599	0.556

Min: minimum; Max: maximum; IQR: Interquartile Range; TAL: total antioxidant level; TOL: total oxidant level; PON; Paraoxonase; SPON: stimulated paraoxonase; ARES: Arylesterase; OXTTL: oxygenated total thiol levels; CRP: C-reactive protein.

**Figure 1:**
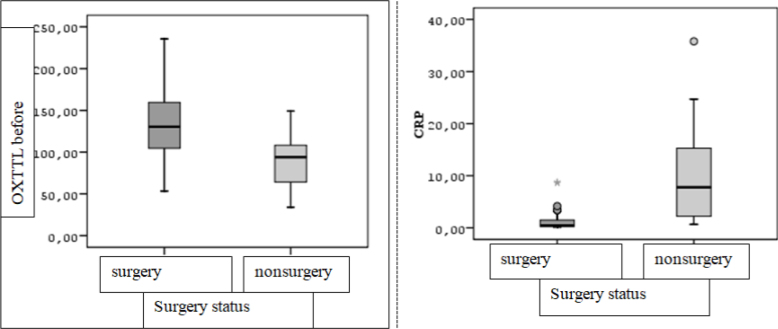
Box-line graph of oxygenated total thiol levels before and C-reactive protein values of individuals according to surgery status.

OXTTL: oxygenated total thiol levels; CRP: C-reactive protein.

The median OXTTL before surgery was statistically significantly higher than the median of those who did not have surgery (Z=2.760, p=0.005, p<0.05). Similarly, the CRP values of individuals differed significantly between the groups (Z=3.697; p<0.001, p<0.05). The median CRP of those who had surgery was 0.48 (IQR=1.26), while the median of CRP was 7.78 (IQR=19.84) for those who could not have surgery (Figure 1).

**Figure 2:**
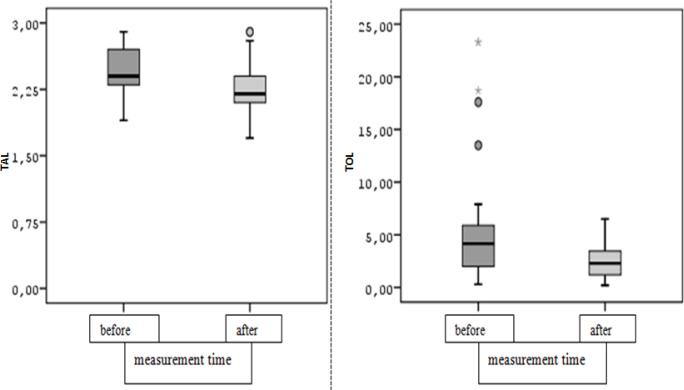
Box-line graph of total antioxidant level and total oxidant level values according to measurement times

TAL: total antioxidant level; TOL: total oxidant level.

The median of the preoperative TAL variable of the individuals was statistically significantly higher than the postoperative value (Z=3.904; p<0.001, p<0.05). The preoperative median TAL was 2.40 (MWG=0.43), while the postoperative median TAL was 2.20 (MAG=0.33) (Table 7). Similarly, the median TOL was found to be statistically significantly lower postoperatively than preoperatively (Z=3.599; p<0.001, p<0.05) (Figure 2).

**Table 7:** Comparison of pre- and post-operative variable values.

(n=34)	Surgery before Median (IQR)	Surgery after Median (IQR)	Z	p
TAL	2.40 (0.43)	2.20 (0.33)	3.904	<0.001
TOL	4.17 (4.09)	2.30 (2.36)	3.599	<0.001
PON	140.60 (112.78)	128.75 (101.63)	4.590	<0.001
SPON	414.80 (376.03)	372.95 (368.38)	3.770	<0.001
ARES	78.90 (38.78)	69.85 (42.60)	2.659	0.008
OXTTL	130.50 (57.33)	119.35 (56.15)	0.162	0.871

IQR: Interquartile Range; TAL: total antioxidant level; TOL: total oxidant level; PON: Paraoxonase; SPON: stimulated paraoxonase; ARES: Arylesterase; OXTTL: oxygenated total thiol levels.

The PON, SPON and ARES values of the patients decreased significantly after the operation (Table 7). OXTTL median decreased after the operation compared to before, but this difference was not statistically significant (Z=0.162; p=0.871, p>0.05).

**Figure 3:**
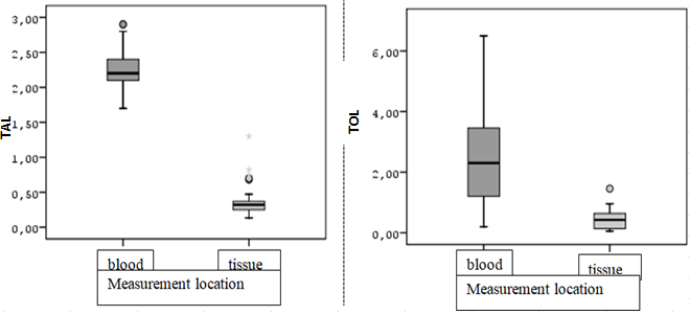
Box-line graph of total antioxidant level and total oxidant level values according to the measurement location.

TAL: total antioxidant level; TOL: total oxidant level.

It was observed that there was a statistically significant difference between the postoperative blood values and tissue values of the patients in terms of all the variables mentioned (Table 8). The TAL and TOL values obtained from the tissue were statistically significantly lower than the values obtained from the blood after surgery (Z=5.086; p<0.001 and Z=4.761; p<0.001, respectively) (Figure 3).

**Table 8:** Comparison of blood values and tissue values in the post-operative variables.

(n=34)	Blood median (IQR)	Tissue median (IQR)	Z	p
TAL	2.20 (0.33)	0.32 (0.13)	5.086	<0.001
TOL	2.30 (2.36)	0.43 (0.52)	4.761	<0.001
PON	128.75 (101.63)	0.42 (0.40)	5.086	<0.001
SPON	372.95 (368.38)	3.17 (2.92)	5.086	<0.001
ARES	69.85 (42.60)	25.72 (13.10)	4.915	<0.001

IQR: Interquartile Range; TAL: total antioxidant level; TOL: total oxidant level; PON: Paraoxonase; SPON: stimulated paraoxonase; ARES: Arylesterase.

**Figure 4:**
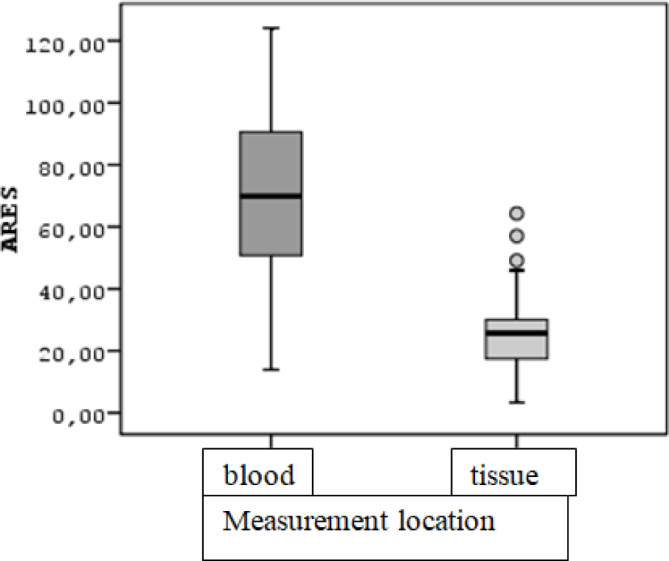
Box-line graph of arylesterase (ARES) variable values according to the measurement location.

The median of ARES obtained from blood is 69.85 (IQR=42.60) and the median of ARES obtained from tissue is 25.72 (IQR=13.10) (Figure 4). The median of ARES values taken from the blood of the patients was significantly higher than the median of the values taken from the tissue (Z=4.915; p<0.001, p<0.05).

It was determined that TAL after, TOL after values did not differ statistically significantly according to operation time (Z=0.509; p=0.631 and Z=1.245; p=0.217, p>0.05, respectively). The TAL post values of those with an operation duration of 30 minutes or less and those with a duration of over 30 minutes were similar (Table 9).

**Table 9:** Comparison of the post-operative variables according to the operation time.

(n=34)	Surgery time		Z	p
	<30 minutes (n=12) Median (IQR)	>30 minutes (n=22) Median (IQR)		
TAL after	2.20 (0.18)	2.25 (0.53)	0.509	0.631
TOL after	2.75 (5.19)	2.05 (2.28)	1.245	0.217
PON after	113.30 (116.60)	137.00 (151.83)	1.081	0.292
SPON after	285.75 (391.88)	397.50 (530.10)	1.225	0.231
ARES after	61.50 (29.08)	75.65 (37.28)	1.730	0.087
OXTTL after	107.95 (37.75)	130.60 (81.43)	1.586	0.118

IQR: Interquartile Range; TAL: total antioxidant level; TOL: total oxidant level; PON: Paraoxonase; SPON: stimulated paraoxonase; ARES: Arylesterase; OXTTL: oxygenated total thiol levels.

While the median of ARES after values of those with 30 min or less operation time is 61.50 (IQR=29.08), the median ARES after value for those with an operation time longer than 30 min is 75.65 (IQR=37.28) (Z=1.730; p=0.087, p>0.05).

## DISCUSSION

Gallbladder stones are a common problem seen in approximately one out of every ten people in society, and, because of its many different complications, it can cause hospital admissions in various forms in emergency or elective conditions^6,19^.

For this reason, many studies have been carried out on the pathophysiology of the disease. However, studies at the cellular level are limited. TOL and TAL markers, which have been accepted as one of the important indicators of cellular activity in recent years, are important in this regard.

In this study, TOL and TAL in blood and tissue samples of 45 randomly selected patients who applied to our clinic were studied, and we tried to show how these levels might change in inflammatory or stress conditions.

Previously, TOL and TAL were compared in relation to various subjects. Bipolar disorder, depression, cirrhosis, type 2 diabetes are just a few of the research topics related to oxidant levels in the literature^1,2,5,8,12,13,15,16,20^.

However, despite our detailed review of the literature, we could not find another study that was similar to our previous study, measuring and comparing TAL and TOL levels in cholelithiasis and its complications. Some studies alone reveal that lipid peroxidation increases in patients with gallstones and that there are fluctuations in these oxidant-antioxidant levels^6,10,14^. However, the operation factor was not evaluated in any of these studies and this situation forms the basis of our studies. In our study, 45 patients who applied to our clinic at different times were randomly selected without determining any exclusion criteria, and no attempt was made to maintain a balance between the groups. For this reason, number differences have emerged among some parameters, which may cause problems in statistical comparisons. However, there is no significant difference between the expected results and the obtained ones. Expected values were determined by considering other studies on this subject^6,14^.

In the study, oxidant parameters were studied in the pre- and post-operative samples of the patients. Thus, we tried to show how these values change when the stress factor comes into play. For this purpose, blood was taken from the patients before and 6 hours after the operation. In studies on other diseases in the literature, control blood was usually taken on the second day of hospitalization^15,16,19^. We, on the other hand, found it appropriate to take control blood at the 6^th^ hour of surgery.

When we look at the demographic distributions in the study, it is remarkable that female patients are more numerous than males. This is consistent with the general population. Hospital admissions due to cholelithiasis and its complications are higher in women than men. Patients are usually 40 years or older (n=33). When evaluated statistically, it was determined that TOL and TAL did not differ significantly between age and gender groups. The information obtained from the literature determined that TAL and TOL values did not differ significantly between genders^9,14^. Although minimal decreases in values are observed with age, these are not statistically significant (p<0.05).

The study divided the patients into two according to whether they had surgery or not. Control blood was not taken from the patients who did not have surgery. Patients with acute cholecystitis and biliary colic and biliary pancreatitis who were followed up with conservative treatment were not found suitable for surgery and were followed up. These patients are usually those who apply to the emergency department with complaints of acute pain, and their inflammatory status is higher than the patients who underwent elective surgery. CRP values are expected to be high in these patients during this process. Therefore, the CRP values studied from the blood taken from these two groups of patients at hospitalization show a significant difference (p<0.001, p<0.05).

When the parameters in the blood samples taken before and 6 hours after the operation in the operated patients were compared, it was observed that the oxidant and antioxidant values decreased significantly in the postoperative period (Z=3.599; p<0.001, p<0.05). As mentioned earlier, this decrease is significant and logical when both groups of free radicals need to be spent in the management process. In another study conducted with data obtained from blood taken before and after blood sugar regulation in patients with type 2 diabetes, it was stated that these values regressed after treatment.

Tissue derived from blood benefits from operated devices has also been studied. This tissue sample is about half a square centimeter piece obtained from the fundus of the gallbladder. Interpretation of oxidative balance in vivo assessments is made using a plasma-derived blanket. The fact that the ratio of oxidative values obtained from the tissue to the amount of blood is quite low in our study is due to the dilution that is technically aimed at ensuring homogeneity in tissue samples. However, when the measured values are examined, it is seen that the oxidative event in the tissue is not observed in the blood values; in other words, it can be interpreted that the disease does not become systemic (p<0.001, p<0.05). The most likely explanation for this is that the patients who were operated on were patients who could not have an acute inflammatory event under elective conditions.

The average duration of gallbladder stone surgeries performed in our clinic is around 30 minutes. Considering this information, the cutoff value is 30 min. The patients who were operated on were divided into two groups. There was no statistically significant difference between TAL and TOL postoperatively between these two groups. This can be interpreted to mean that the oxidant level of the operation period depends, in the early period, on the stress factor (Z=1.730; p=0.087, p>0.05).

It is not possible to compare the data we have obtained since there is no study in the literature for patients who underwent surgery for similar reasons. Although it is a common disease in the population, it can be thought that the number of volunteers in the study was small. An important reason for this low number is that the study can be performed by a single person with the close course of the patient in the clinic. Patients whose data could not be completed due to some disruptions in hospitalization, follow-up, or acquisition of surgical tissues were excluded from the study.

Nevertheless, the data of our study show similarities with studies conducted for other purposes and measuring oxidant levels^13,15,20^. For example, the median TAL in operated patients was found to be 2.4, and this value was found to be 2.34 in another study conducted with blood obtained from patients hospitalized for myocardial infarction (n=58). This minimal regression is likely due to the higher mean age of patients hospitalized for myocardial infarction^21^.

The oxidant and antioxidant parameters do not differ with factors such as age, gender, and operation time. While these values decreased significantly in the postoperative period, a general regression was observed in the sub-data constituting these values.

The usability of these data in clinical daily use in the follow-up of these diseases is a complete mystery. The data we have are insufficient to decide on this issue, and many more experimental studies on this subject are required. The implementation of these tests today is still difficult, time-consuming, and costly. Considering that the disease is common, the test to be used in the follow-up of treatment success in cholelithiasis is applied quickly, just as in other common diseases. For this reason, although these parameters do not seem appropriate as of today in the evaluation of cholelithiasis and its associated symptoms, it is obvious that they are open to developments.

## CONCLUSIONS

The following conclusions can be drawn from this study:

1. TOL and TAL do not differ according to age and gender. That is, when evaluating these values, separate classifications will not be required.
2. After the treatment, a significant regression was observed in these levels in the patients. However, this decline may also be due to consumption in the current process. It is not possible to make a full distinction.
3. The use of these tests for follow-up or screening purposes in the clinic does not seem logical today, considering the frequency of the disease, due to the expensive and long-term results.
